# A Narrow Dual-Band Monolayer Unpatterned Graphene-Based Perfect Absorber with Critical Coupling in the Near Infrared

**DOI:** 10.3390/mi11010058

**Published:** 2020-01-01

**Authors:** Pinghui Wu, Zeqiang Chen, Danyang Xu, Congfen Zhang, Ronghua Jian

**Affiliations:** 1 Research Center for Photonic Technology, Fujian Key Laboratory for Advanced Micro-Nano Photonics Technology and Devices & Key Laboratory of Information Functional Material for Fujian Higher Education, Quanzhou Normal University, Quanzhou 362000, China; phwu@zju.edu.cn (P.W.); czqchem@qztc.edu.cn (Z.C.); 2 College of Science, Zhejiang University of Technology, Hangzhou 310023, China; xudanyang@zjut.edu.cn; 3 College of Life Science and Engineering, Southwest University of Science and Technology, Mianyang 621010, China; Zhang2017@swust.edu.cn; 4 School of Science, Huzhou University, Huzhou 313000, China

**Keywords:** graphene, near infrared, critical coupling, dual-band perfect absorption

## Abstract

The combination of critical coupling and coupled mode theory in this study elevated the absorption performance of a graphene-based absorber in the near-infrared band, achieving perfect absorption in the double bands (98.96% and 98.22%), owing to the guided mode resonance (the coupling of the leak mode and guided mode under the condition of phase matching, which revealed 100% transmission or reflection efficiency in the wavelet band), and a third high-efficiency absorption (91.34%) emerged. During the evaluation of the single-structure, cross-circle-shaped absorber via simulation and theoretical analysis, the cross-circle shaped absorber assumed a conspicuous preponderance through exploring the correlation between absorption and tunable parameters (period, geometric measure, and incident angle of the cross-circle absorber), and by briefly analyzing the quality factors and universal applicability. Hence, the cross-circle resonance structure provides novel potential for the design of a dual-band unpatterned graphene perfect absorber in the near-infrared band, and possesses practical application significance in photoelectric detectors, modulators, optical switching, and numerous other photoelectric devices.

## 1. Introduction

After the successful preparation of monolayer graphene from experiments through A.K. Geim’s group using a mechanical stripping method in 2004, its unique electronic band structure has attracted wide attention from scholars [1,2,3,4,5,6,7,8]. As a revolutionary two-dimensional material, graphene has an ultra-broad spectral response range (from ultraviolet to terahertz) [1], ultra-thin atomic layer thickness (the monoatomic layer thickness is merely 0.34 nm), and ultra-high carrier mobility (approximately 200,000 cm^2^∙V^−1^∙s^−1^) [9,10,11], making it an ideal material for designing optoelectronic devices. However, the monolayer graphene performs poorly at absorbing light in the visible and near infrared bands, with a mere 2.3% [12,13,14], which limits the quantum efficiency and application in optoelectronic devices. Fortunately, researchers have confirmed that graphene supports surface plasmon resonance (SPR) in the far infrared and terahertz bands [15,16,17,18,19,20,21], enhancing the absorption and broadening the application scope of graphene in optoelectronic and photoelectric devices, such as in modulators, filters, photoelectric detectors, optical switches, and biosensors [22,23,24,25,26,27,28,29]. Correspondingly, the lack of diversity of graphene-based photoelectric devices in the visible and near-infrared regions has received more urgent attention. Due to the current limited doping level, graphene barely supports surface plasmon resonance in the visible and near-infrared bands, meaning it is difficult to achieve efficient light absorption.

In recent years, scholars have proposed various measures to improve the absorption of graphene in the visible and near-infrared range, commonly by coupling graphene with some resonance structures [30,31,32]. For instance, a polarization-dependent graphene absorber with a one-dimensional, sub-wavelength dielectric grating has been theoretically proved to be capable of attaining perfect absorption [33]. It has been confirmed that a sub-wavelength structure based on graphene, in TE (the direction of electric field is parallel to *y*) polarization mode, displays a peak absorption over 99% [34] at a wavelength of about 1500 nm, which explains that a light source with a polarization-independent absorption structure is preferential under normal incident conditions with the purpose of satisfying the application requirements. Piper and Fan demonstrated single-layer graphene with a two-dimensional photonic crystal plate having a high refractive index exhibits perfect absorption in the near-infrared band [35], where the coupled mode theory (CMT) expounds the critical coupling phenomenon [36]. Also, coupling monolayer graphene with a two-dimensional photonic crystal plate on the top of back metal mirror provides an absorption of up to 77% [37]. When the photonic crystal plate with a high contrast refractive index forms an optical resonator in the absorber, the resonant mode is sensitive to the parameter changes and is well-confined to the plate, which creates the perfect absorption; however, the complex preparation process means it is difficult to make such an theoretical construction a reality for experiments because the fabrication of high-refractive-index contrast structures currently requires multiple layers of dielectrics to create Bragg mirrors. Moreover, most of the graphene perfect absorbers in the visible and near-infrared bands have only one resonance mode. A few absorbers that perform dual-band absorption by blocking the transmission of incident light through heaping multi-layer dielectrics is possible, but it requires a demanding preparation method with a complex process.

In this paper, we propose a cross-circle-shaped dual-band monolayer unpatterned graphene-based perfect absorber composed of Au, SiO_2_, and a two-dimensional polymethyl methacrylate (PMMA) layer with a cross-circle-shaped air groove. From numerical simulations and theoretical analysis, the crucial relationship between tunable parameters and absorption performance was investigated, along with a brief presentation of the quality factor and universality. The simulation results proves that a graphene perfect absorber achieved a double-band perfect absorption (98.96% and 98.22%) based on guided-mode resonance (GMR) and critical coupling, and possessed a third high-efficiency absorption (91.34%). Simultaneously, the structure had no polarization dependence or angle sensitivity. Compared with most of the structures studied, our structure has a simpler fabrication process and a larger absorption band.

## 2. Geometric Structure of Model and Method

**Figure 1:**
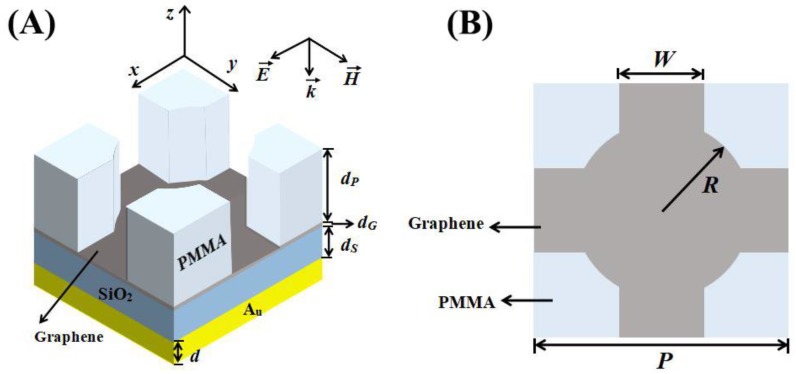
(**A**) Three-dimensional schematic diagram of the structure of the cross-circle-shaped periodic monolayer graphene unpatterned perfect absorber. The graphene layer was sandwiched between polymethyl methacrylate (PMMA) and SiO_2_ layers. The fundamental parameters were: *d_P_* = 330 nm, *d_S_* = 240 nm, and *d* = 200 nm. (**B**) The corresponding top view with the specific geometric parameters shown in the figure. The fundamental parameters were: *W* = 400 nm, *R* = 500 nm, and *P* = 1250 nm.

The following introduces the cross-circle periodic monolayer graphene unpatterned perfect absorber with specific parameters. As shown in Figure 1A, the three-dimensional diagram displays the frame structure of the whole absorber, with the vertical view displaying the parameter details given in Figure 1B. Graphene was placed between the PMMA layer with a cross-circle-shaped air groove and a SiO_2_ layer, which formed a sandwich stacking structure similar to a square. At the bottom of the absorber, the metal mirror (gold layer) reduced the transmission of light, where Au was chosen due to the parameters from the Drude model, *ε_∞_* = 1.0, *ω_p_* = 1.37 × 10^16^ S^−1^, and *γ* = 8.17 × 10^13^ S^−1^ [38]. In the simulation, the thickness of the unpatterned monolayer graphene was *d_G_* = 0.34 nm [9], whose refractive index followed the expression *n* = 3 + 5.446j × *λ*/3 μm^−1^, which was fitted using experimental data [39]. Furthermore, the refractive indices of PMMA, SiO_2_, and the cross-circle shaped air groove were 1.48, 1.45, and 1, respectively. Exploring the absorber’s performance with the parameter configurations produced a critical coupling. The fundamental parameters were as follows: thickness of the PMMA layer *d_P_* = 330 nm, thickness of the SiO_2_ layer *d_S_* = 240 nm, thickness of the gold layer *d* = 200 nm, the width *W* = 400 nm, the radius *R* = 500 nm, and the period *P* = 1250 nm. Furthermore, the incident light was *θ* = 0° in TM (the direction of the electric field was parallel with *x*) polarization mode. The numerical simulation analysis depended on the finite difference time domain (FDTD) method [40], where the periodic boundary conditions were chosen to be parallel to the *x* and *y* directions, and the perfect matching layer (PML) was added perpendicular to the *z* direction.

Coupled mode theory (CMT) describes the input–output characteristics of a resonator [41], which covers the direct channel and indirect channel, straightaway reflecting the structure of the channel corresponding to the thin-film material, with the contained resonance excitation, while the indirect channel consists of resonance excitation and coherent interference between two channels, which explains the absorption increment and inhibition. The single resonance mode of the resonator at *ω*_0_ interacts with the incoming wave (amplitude *u*, power *u*^2^) and the outgoing wave (amplitude *y*). Furthermore, the stored energy is *a*^2^. Distinguishing the material’s presence in the cavity by the inherent loss rate *δ* and leakage rate *γ_e_* [35]:

(1) $$\dot{a}=(j\omega_{0}-\gamma_{e}-\delta )a+\sqrt{2\gamma_{e}}u,$$

(2) $$y=\sqrt{2\gamma_{e}}a-u.$$

Therefore, the reflection coefficient of the system can be expressed by the following formula:

(3) $$\Gamma =\frac{y}{u}=\frac{j(\omega -\omega_{0})+\delta -\gamma_{e}}{j(\omega -\omega_{0})+\delta +\gamma_{e}}$$

and the optical absorption is calculated using *A* = 1 − |*Г*|^2^ [42] to give:

(4) $$A=\frac{4\delta \gamma_{e}}{{\left(\omega -\omega_{0}\right)}^{2}+{\left(\delta +\gamma_{e}\right)}^{2}}.$$

When the system is in resonance, *ω* = *ω*_0_ (or in other words, *γ_e_* = *δ*) and the reflection coefficient disappears, meaning the total incident power is absorbed; the system has reached the critical coupling state, hence *A* = 1 in Equation (4), achieving the perfect absorption of the resonator in theory.

## 3. Simulation Results and Discussion

**Figure 2:**
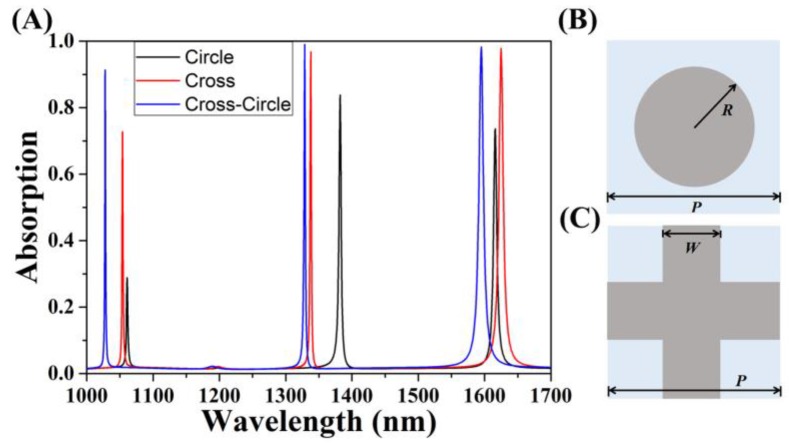
(**A**) Absorption of the cross, circle, and cross-circle-shaped single-structure graphene absorbers. (**B**) Top view of the circle-shaped single-structure graphene absorber with a radius *R*. (**C**) Cross-shaped single-structure graphene absorber with a width *W*. The periods of the above two structures were *P*.

Figure 2A gives the absorption spectra of the cross-circle graphene absorbers and single-structure absorbers at a normal incidence. Undoubtedly, the cross-circle absorber significantly improved the absorption of graphene in the near-infrared region. The mode of *λ* = 1328.42 nm (GMR_0_) and *λ* = 1594.98 nm (GMR_1_) gave the two near-perfect absorption peaks reaching 98.96% and 98.22%, respectively, and the third absorption peak, which reached 91.34% at *λ* = 1027.95 nm (GMR_2_). Owing to the periodic super units constituting the patterned PMMA layer, when normal incident light was coupled into the structure, the patterned PMMA plate could support an in-plane guiding mode, which is usually strongly limited by the PMMA without coupling with external radiation. The cross-circle resonator had the advantages of absorption in each response region and a relative dominance for the absorption peak half-width (FWHM). Specifically, for the GMR_0_ mode, the FWHM scarcely occupied ∆*λ*_0_ = 1.9 nm, while the quality factor *Q*, defined as *Q*_0_ = *λ*_0_/∆*λ*_0_, approached about 699.17, which indicated an extremely narrow linewidth of the spectral selective absorption and high-quality performance. However, the FWHM of the circle-shaped absorber was ∆*λ*_0_ = 4.5 nm and *Q* = 295.19 at *λ* = 1382.39 nm, while the cross-shaped had a ∆*λ*_0_ = 2.4 nm and *Q* = 557.37 at *λ* = 1337.69 nm. According to the CMT, the *Q_δ_* = *ω*_0_/2*δ* and *Q_γe_* = *ω*_0_/2*γ_e_* represent the inherent loss and external leakage of the GMR_0_ mode, respectively. Therefore, we can clearly understand the theoretical quality factor computed from *Q*_CMT_ = (*Q_δ_* × *Q_γe_*)/(*Q_δ_* + *Q_γe_*). When critical coupling occurs, *Q_δ_* and *Q_γe_* are equal. Hence, the above formula simplifies to Q_CMT_ = *Q_δ_*/2 = *ω*_0_/4*δ*, and the parameters are fixed at *δ* = *γ_e_* = 0.507 × 10^12^ Hz. Then, the value of *Q*_CMT0_ was 699.67 (almost identical to *Q*_0_), which indicated that the total absorption could be attributed to critical coupling. Similarly, for GMR_1_ mode, the FWHM was ∆*λ*_0_ = 8.38 nm with *Q*_1_ = 190.33. Therefore, the value of *Q*_CMT1_ was 190.37 (*δ* = *γ_e_* = 1.552 × 10^12^ Hz). This proved that the cross-circle periodic monolayer graphene-based perfect absorber proposed in this paper has a greater research value and practical application in terms of light absorption performance.

**Figure 3:**
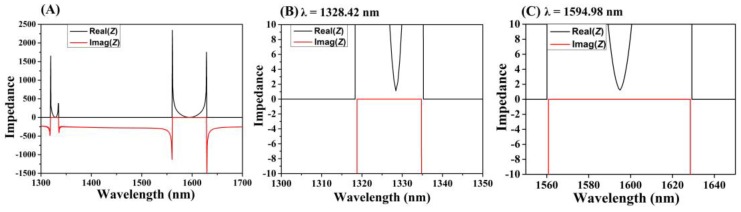
(**A**) Effective impedance diagram of the cross-circle monolayer graphene unpatterned perfect absorber. (**B**) Effective impedance amplification at 1328.42 nm. (**C**) Effective impedance amplification at 1594.98 nm.

Figure 3A exhibits the functional relationship between the system’s effective impedance and the incident wavelength obtained from the impedance equation [43]:

$$Z=\sqrt{\frac{{\left(1+S_{11}\right)}^{2}-S_{21}^{2}}{{\left(1-S_{11}\right)}^{2}-S_{21}^{2}}}$$

The *S*_11_ and *S*_21_ are the scattering parameters relevant to the reflectance and transmittance, respectively. From the point of impedance matching, when the periodicity of the PMMA layer makes the effective impedance of the system match with the free space impedance, the reflection can be effectively suppressed to generate a guided resonance, and the perfect absorption appears [44]. According to the relationship demonstrated in Figure 3B,C, it was confirmed that the cross-circle-shaped graphene absorber absorbed up to 98.96% and 98.22% of the incident light in dual-band because of the critical coupling state.

We studied the internal impedance of two bands when the optical absorption approached the ideal perfect absorption. The impedance increased remarkably after *λ* = 1320 nm and *λ* = 1560 nm for the two bands, respectively. Combining with the absorption spectrum, the reflection of the cross-circle graphene absorber effectively existed in an off-resonant range, resulting in the extremely weak optical absorption. When the system impedance was close to the air impedance in the free space, the reflectivity substantially decreased, leading to the absorption increasing significantly. For the GMR_0_ mode, the impedance was *Z*_0_ = 1.109 − 0.0073i at the resonance wavelength *λ* = 1328.42 nm shown in Figure 3B, which was basically equal to the free space impedance. For the GMR_1_ mode, the impedance was *Z*_0_ = 1.225 − 0.0173i at *λ* = 1594.98 nm, as shown in Figure 3C. Therefore, the absorber was proven to be in a critical coupling state in the dual-band absorption range.

**Figure 4:**
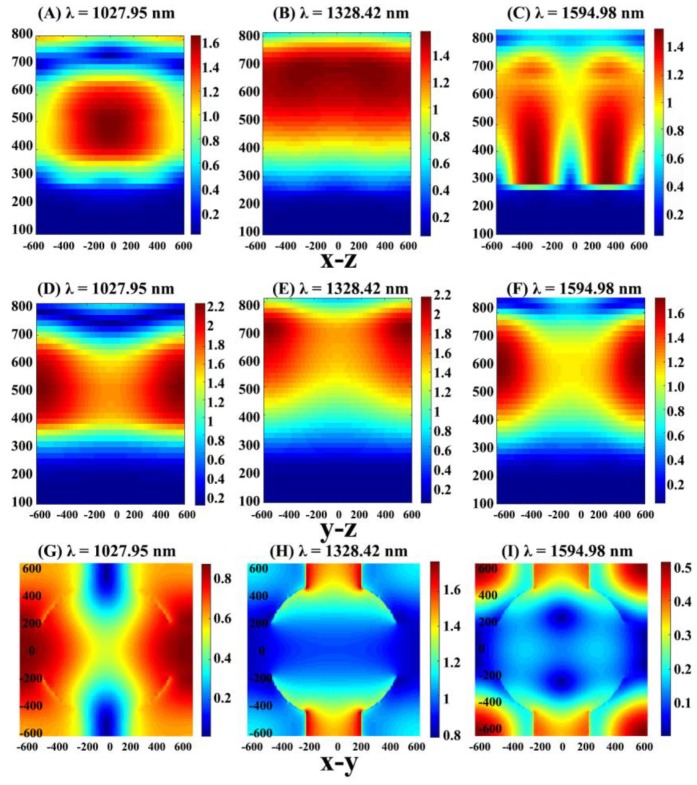
(**A**–**C**) are electric field maps at 1027.95 nm, 1328.42 nm, and 1594.95 nm, respectively. They are all *x*-*z* sectional views. (**D**–**F**) are three electric field maps at 1027.95 nm, 1328.42 nm, and 1594.95 nm, respectively. They are all cross-sectional views in the *y*-*z* direction. (**G**–**I**) are three electric field maps at 1027.95 nm, 1328.42 nm, and 1594.95 nm, respectively. They are all cross-sectional views in the *y*-*z* direction.

In order to have a qualitative understanding of the enhanced performance effect due to critical coupling, the electric field distributions at three resonance peak wavelengths are shown in Figure 4. Three resonance modes were in different positions in the system. Here, this section focuses on two absorption bands that achieved a critical coupling state. The absorption peaks at 1594.98 nm and 1328.42 nm corresponded to different GMR orders: the first order GMR at 1594.98 nm (GMR_1_) and the zeroth order GMR at 1328.42 nm (GMR_0_). The conduction mode captured the incident field around the graphene sheet, which more effectively consumed the local electromagnetic field to boost the absorption, and further generated the resonance absorption effect. Taking the GMR_0_ model as an example, the typical standing wave profile was formed along y direction of the periodic model, which indicated the existence of a GMR. Hence, a GMR could be successfully excited when the phase matching between the incident wave and the leaky lateral waveguide modes was satisfied [45].

**Figure 5:**
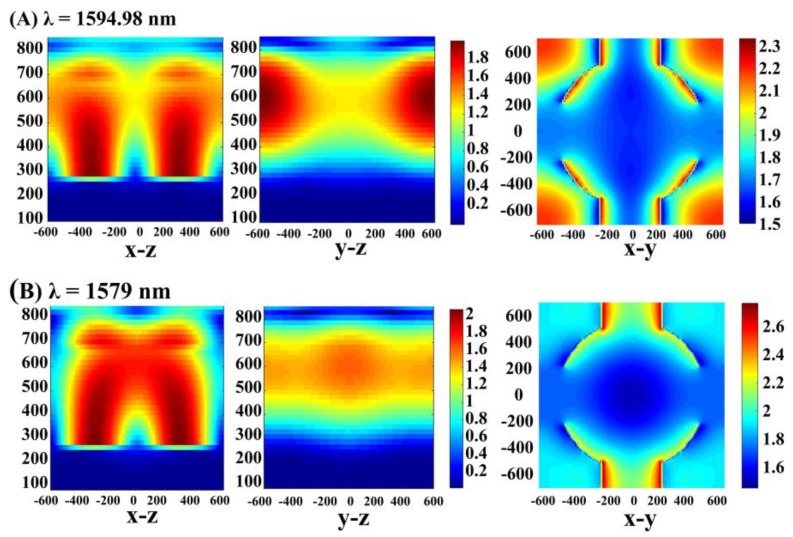
(**A**) Section view of a side electric field under a critical coupling state and the *x*-*y* plane electric field diagram (wavelength: 1594.98 nm). (**B**) Sectional view of a side electric field and the *x*-*y* plane electric field diagram in a non-critical coupling state (wavelength: 1579 nm).

Furthermore, the lateral electric field diagram, which represents the critical coupling and non-critical coupling of the identical resonance mode, was compared and analyzed to understand the critical coupling caused by the guided mode resonance and photonic band gap effect of the photonic crystal layer. We plotted the electric field intensity distribution, where the *x*-*z*, *y*-*z*, and *x*-*y* directions at resonant (1594.98 nm) and off-resonant (1579 nm) wavelengths are shown in Figure 5. As shown in the figure, the electric field intensity around the graphene increased significantly, while the resonator was not stimulated. When the system was off-resonant, the reflection coefficient was equal to 1, which corresponds to the low absorption (1579 nm) in Figure 2A, and the electric field intensity distribution emerged as given in Figure 5B. Comparing the *y*-*z* electric field patterns in resonance with that in off-resonance, it was found that the strong electric field in resonance was principally distributed at both ends of the y direction to form the standing wave mode, while the integral electric field lacked intensity in off-resonance. From the x-y pattern of the upward system side, the intensity at 1594.98 nm primarily concentrated around the resonator, and the electromagnetic field of the incident at the center was absorbed by graphene because the GMR consumed it better. The *x*-*y* directional electric field at 1579 nm showed that the incident field in the resonator was incompletely consumed, and hence the absorption performance was distinctly weaker than that at 1594.98 nm.

**Figure 6:**
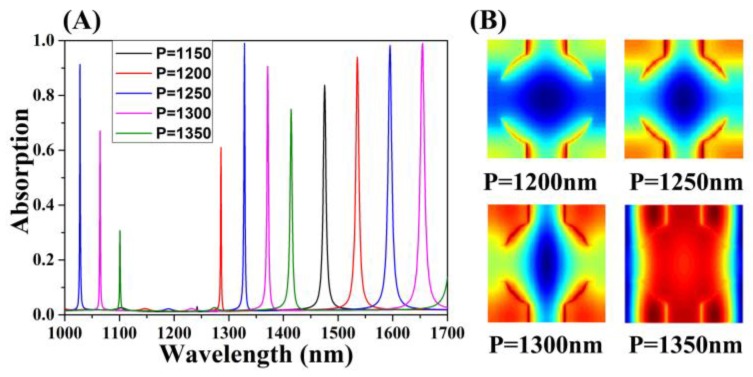
Changes in absorption while varying the period *P* of the absorber structure and keeping the other parameters unchanged. (**A**) When *P* increased from 1150 nm to 1350 nm, the corresponding optical absorption was obtained. (**B**) When the structure had a different period *P*, the electric field map corresponding to the graphene layer was obtained.

Parameters except the period *P* were left unchanged using a control variable method. Considering that the monolayer graphene absorption in the near-infrared range is largely independent of frequency and the correspondingly settled intrinsic loss rate *δ*, resulting in controlling the leakage rate *γ_e_* of structure more accurately to obtain the perfect absorption of graphene. First, we provide the relationship between period *P* and leakage rate *γ*_e_ by displaying the effect of the variable period on the absorption performance. When the period *P* of the cross-circle absorber changed, the absorption peak transformed significantly, as shown in Figure 6A, where the absorption spectra variations of the three guided mode resonances were generally coincident. For the GMR_0_ mode, when the period *P* broadened from 1150 nm to 1350 nm, the corresponding optical absorption increased to 98.96% (*P* = 1250 nm), and then descended. During the process, the leakage rate *γ*_e_ of the resonator increased, and the trend of the optical absorption, which represents the system structure, experienced three states: uncoupled, critically coupled, and over-coupled. Under the critical coupling state, the maximum ground light absorption was enhanced to achieve the perfect optical absorption of graphene. Synchronously, the corresponding absorption spectra red-shifted significantly, where the absorption peak shifted from 1241 nm to 1423 nm. Figure 6B reveals the electric field distribution in the graphene layer under different period conditions. When the structure was uncoupled, the electric field intensity at the four corners was exceedingly weak, but the intensity increased due to a weak resonance at the two ends of the y direction alone, resulting in definitively lower absorption. When the structure was over-coupled, most of the incident light field was not caught by the guided mode concentrate in the resonator center leading to the imperfect absorption.

**Figure 7:**
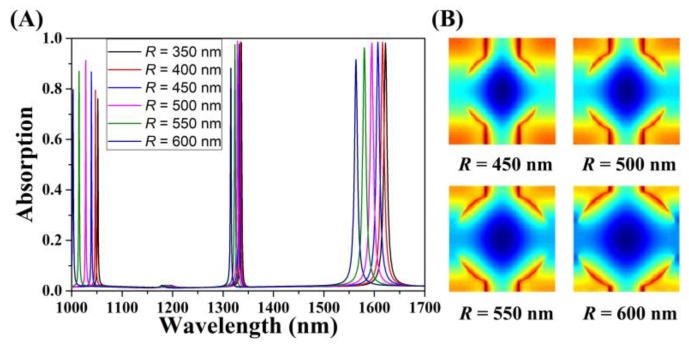
Changes in absorption while varying the radius *R* of the absorber structure and keeping the other parameters unchanged. (**A**) Optical absorption when *R* increased from 350 nm to 600 nm. (**B**) The electric field diagram for the graphene layer with varying radius *R*.

**Figure 8:**
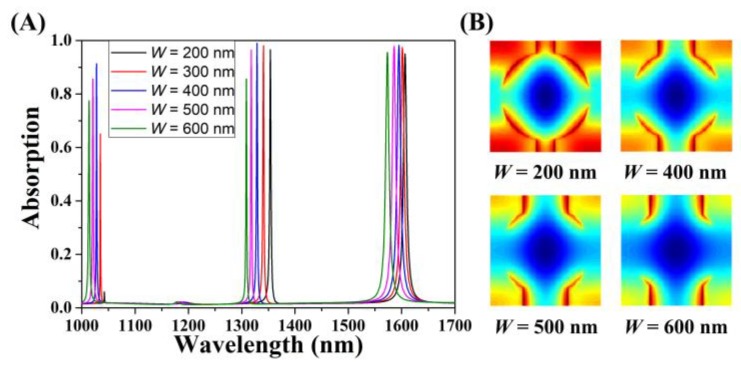
Changes in absorption while varying the width *W* of the absorber structure and keeping the other parameters unchanged. (**A**) Optical absorption when *W* increased from 200 nm to 600 nm. (**B**) The electric field diagram for the graphene layer with varying width *W*.

Next, the geometric properties (radius *R* and width *W*) of the cross-circle structure were changed; the following discusses the influence of the changes on the optical absorption performance. The absorption spectra are shown in Figure 7A and Figure 8A for changing *R* and *W*, respectively. In terms of radius *R*, with an increase of the radius *R*, the absorption spectra of the three resonance modes had the similar trends: the light absorption first increased and then decreased. When the radius *R* increased from 350 nm to 500 nm, the absorption peak also increased with a small increment. When *R* = 500 nm, the absorption peak reached the maximum. When *R* was greater than 500 nm, the absorption decreased significantly, until *R* = 600 nm, where the absorption decreased to 90%. Meanwhile, the absorption spectrum peak of graphene monolayer showed a linear blue shift, which was due to the change of the structure’s gap position. Among the three modes, the blue-shift degree of GMR_0_ mode was the smallest, and the blue-shift degree of the GMR_1_ mode was the most obvious. Figure 7B shows the electric field intensity distribution of the graphene layer of the system when different radii were used. It can be seen that there was no significant difference in the intensity between the two electric field diagrams with *R*s of 450 nm and 500 nm. Only at *R* = 600 nm did the electric field intensity at the four corners of the structure weaken significantly. The above values indicated that the influence of the radius *R* on the external leakage rate of the resonator was small, and the influence on the GMR_1_ mode was slightly stronger than that on the GMR_0_ mode. Under certain circumstances, it can be considered that when *R* = 400 nm, 450 nm, or 500 nm, both resonance modes could achieve the critical coupling state of guided mode resonance of the system and enhance the light absorption of graphene. For practical applications, radius parameter adjustment can provide more convenience.

The relationship between width *W* and leakage rate *γ_e_* of the cross-circle-shaped absorber and its influence on the absorption performance are discussed here. As shown in Figure 8A, the trend of the absorption of three modes was consistent with the broadening of the width *W*. Taking GMR_0_ as an example, when the width *W* broadened from 200 nm to 600 nm, the corresponding optical absorption first increased and then decreased. Critical coupling emerged at *W* = 400 nm, which achieved high-efficiency (98.96%) absorption. Up until *W* = 600 nm, the optical absorption decreased to 84.85%, which accords with the three stages of the coupling process. With the broadening of *W*, the absorption spectra exhibited a clear blue shift. Comparatively speaking, the blue shift of GMR_0_ was the most conspicuous among the three models, which was different from circumstances mentioned in the last paragraph, indicating that width *W* had a minuscule influence on the *γ_e_* of the structure according to the coupled mode theory with a guided resonance formalism [46]. Within a wide parameter range of *W*, the absorber attained more than 95% absorption, which means that the width *W* has a variety of selectivity. Under the uniform high-efficiency absorption conditions, the specified spectral response could be achieved by selecting parameters appropriately, which provides a more flexible choice for the absorber practical applications in optoelectronic devices.

**Figure 9:**
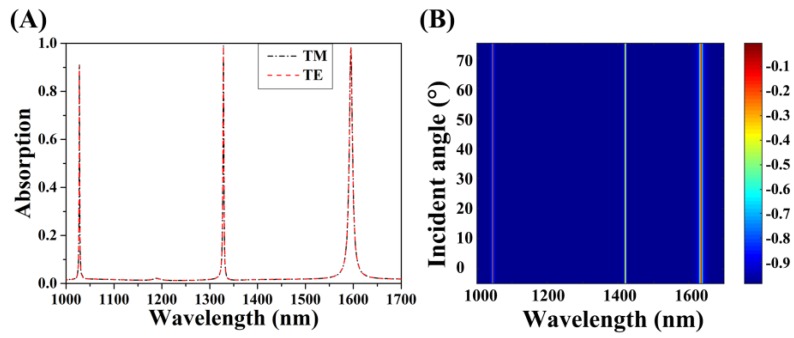
Changes in absorption while varying the angle or polarization of the incident light and keeping the other parameters unchanged. (**A**) The optical absorption of the absorber in TE (the direction of electric field is parallel to *y*) and TM (the direction of electric field is parallel to *x*) polarization modes. (**B**) Varying the incident angle when the incident light source was in TM polarization mode.

Based on the incident source, the polarization dependence and angle sensitivity of the absorber was explored in terms of the polarization mode and incident angle. As shown in Figure 9A, the identical absorption (including the absorption peak, resonance peak wavelength, and the FWHM) of the cross-circle graphene absorber in two incident light polarization modes (TM polarization and TE polarization) is provided. The structure had no dependence on the incident polarization. Thus, we merely provide the absorption obtained by changing the incident angle *θ* under TM polarization, as shown in Figure 9B. When *θ* increased, the wavelength of the three absorption peaks almost remained unchanged, and the absorption intensity changed inconspicuously, which indicated the structure was angle insensitive. Because the guided mode resonance was insensitive to the incident angle [34,47,48,49,50], this provides useful application value in integrated optoelectronic devices. Therefore, we proved that the designed structure could simultaneously achieve critical coupling of multiple resonances, which is the main technical index of multispectral optical detection.

**Figure 10:**
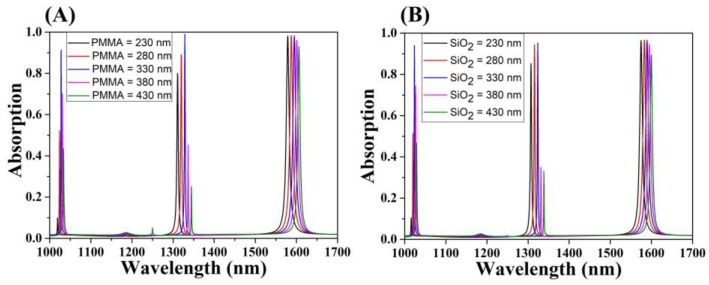
(**A**) Change in the absorption of the PMMA layer with varying incident wavelength. (**B**) Change in the absorption with varying incident wavelength after the PMMA layer was replaced by a SiO_2_ layer.

We intended to explore whether the presented model was universal. The thickness of the PMMA layer (*d_p_*) was changed to investigate the effect of the photonic crystal layer on the absorber absorption performance, as shown in Figure 10A. Compared with the two parameters (*R* and *W*), the thickness of PMMA layer had a minor effect on graphene, which included the insignificant change of the peak absorption and the negligible red shift of resonance peak wavelength, because the leakage rate was insensitive to its small variation. For the GMR_0_ mode, the peak absorption decreased significantly when *d_P_* continued to increase from 330 nm to 430 nm. Meanwhile, the general applicability of the structure was studied by replacing the PMMA layer with a SiO_2_ layer. In Figure 10B, the thickness of SiO_2_ layer was adjusted to provide the same thickness variation as the PMMA layer. Three guided mode resonances also appeared. When the thickness of the SiO_2_ layer increased from 230 nm to 430 nm, the absorption peak increased first and then decreased. When the thickness of the SiO_2_ was 330 nm, the maximum absorption at *λ* = 1323.59 nm was 95.20%, which can be considered as the perfect absorption state. The absorption wavelength red-shifted slightly, which was extremely similar to the situation with a changing *d_p_*. By adjusting the *d_p_*, the spectral selectivity of the structure was improved, which showed the experimental feasibility for the design of this paper. Meanwhile, the excellent absorption obtained by substituting the SiO_2_ layer in for the PMMA layer proved that the absorber has a universal applicability in the selection of photonic crystal layer materials to provide more feasible design approaches for practical applications.

## 4. Conclusions

In conclusion, the periodic monolayer graphene unpatterned perfect absorber with a cross-circle shaped groove air resonator provided critical coupling with the guided and leakage modes at *λ* = 1328.42 nm and *λ* = 1594.98 nm, thus achieving double-band perfect absorption (98.96% and 98.22%), with a third absorption (91.34% at *λ* = 1027.95 nm) reaching a high efficiency. Moreover, the structure possessed excellent quality factors (699.17, 190.33, and 708.93). The simulation results demonstrated that the absorption spectrum, which exhibited variation in the absorption performance in terms of achieving the coupling process, could be actively selected by adjusting the geometrical parameters of the absorber to influence the system leakage rate *γ_e_*. Meanwhile, the absorber had no polarization dependence nor angle sensitivity due to the insensitivity of the guided mode resonance toward the incident angle. After replacing the photonic crystal layer with a material with a similar refractive index, the negligible absorption variation and high efficiency absorption showed that the structure had universal applicability. Therefore, the cross-circle-shaped absorber has broad application prospects in the design and manufacture of photoelectric devices, such as photoelectric detectors, chemical sensors, optical switches, modulators, and so on, which provides new inspiration and ideas for the design of near-infrared tunable graphene multi-band perfect absorption materials.
